# MicroRNAs in Transforming Growth Factor-Beta Signaling Pathway Associated With Fibrosis Involving Different Systems of the Human Body

**DOI:** 10.3389/fmolb.2021.707461

**Published:** 2021-07-26

**Authors:** Xiaoyang Xu, Pengyu Hong, Zhefu Wang, Zhangui Tang, Kun Li

**Affiliations:** Department of Oral and Maxillofacial Surgery, Xiangya Stomatological Hospital and School of Stomatology, Central South University, Changsha, China

**Keywords:** TGF-β, signaling pathway, miRNA, mechanism, fibrosis

## Abstract

Fibrosis, a major cause of morbidity and mortality, is a histopathological manifestation of many chronic inflammatory diseases affecting different systems of the human body. Two types of transforming growth factor beta (TGF-β) signaling pathways regulate fibrosis: the canonical TGF-β signaling pathway, represented by SMAD-2 and SMAD-3, and the noncanonical pathway, which functions without SMAD-2/3 participation and currently includes TGF-β/mitogen-activated protein kinases, TGF-β/SMAD-1/5, TGF-β/phosphatidylinositol-3-kinase/Akt, TGF-β/Janus kinase/signal transducer and activator of transcription protein-3, and TGF-β/rho-associated coiled-coil containing kinase signaling pathways. MicroRNA (miRNA), a type of non-coding single-stranded small RNA, comprises approximately 22 nucleotides encoded by endogenous genes, which can regulate physiological and pathological processes in fibrotic diseases, particularly affecting organs such as the liver, the kidney, the lungs, and the heart. The aim of this review is to introduce the characteristics of the canonical and non-canonical TGF-β signaling pathways and to classify miRNAs with regulatory effects on these two pathways based on the influenced organ. Further, we aim to summarize the limitations of the current research of the mechanisms of fibrosis, provide insights into possible future research directions, and propose therapeutic options for fibrosis.

## Introduction

Fibrosis is a histopathological manifestation of many chronic inflammatory diseases (Wynn, 2007). Some fibroproliferative diseases, such as progressive kidney disease, hepatitis, pulmonary fibrosis, cardiovascular disease, scleroderma, and systemic sclerosis, eventually cause high morbidity and mortality by influencing diverse systems of the human body (Wynn, 2007). Fibrosis is characterized by the undue aggregation of extracellular interstitial constituents in and around injured or inflamed tissues, such as collagen and fibronectin (Wynn, 2008). If the causative disease continues to progress, fibrosis leads to collapse of tissue structure, loss of organ function, and finally death. Factors causing fibrotic diseases are genetic, inflammatory, environmental, etc. (Wynn, 2008) Research is underway for the molecular mechanism of fibrotic diseases and valid treatments to prevent or reverse the progression of these diseases (Wynn, 2008; Meng et al., 2016).

Transforming growth factor beta (TGF-β), a key molecule in the development of fibrosis, can regulate a series of biological processes, including the cell growth cycle, cell differentiation, immune regulation, and extracellular matrix (ECM) deposition (Ikushima and Miyazono, 2012; Meng et al., 2016). Two types of TGF-β signaling pathways regulate fibrosis: the canonical signaling pathway, represented by SMAD-2/3, and the noncanonical pathway, which does not involve SMAD-2/3 participation (Meng et al., 2016; Finnson et al., 2020). Increasing evidence confirms that the pathogenesis of fibrosis includes the canonical TGF-β signaling pathway and abnormal activation of the noncanonical TGF-β pathway (Finnson et al., 2020). Therefore, further research of the molecular mechanism of these signaling pathways regulating fibrotic diseases and potential molecular targets for treatments is warranted in the future.

MicroRNA (miRNA), a type of non-coding single-stranded small RNA, comprises approximately 22 nucleotides encoded by endogenous genes, which can regulate physiological and pathological processes in fibrotic diseases (Liu et al., 2018). Its central role in the research of fibrotic disease treatment and pathogenesis has garnered widespread attention in recent years (Banerjee et al., 2011; Liu et al., 2018; Li et al., 2019). Accumulating evidence confirms that miRNAs, such as miR-125a-5p, miR-29a-3p, miR-181a-5p, and miR-216a, can positively and negatively regulate the canonical TGF-β/SMAD pathway to affect myofibroblast transformation, collagen synthesis, and ECM deposition in fibrotic diseases (Yang et al., 2016; Henry et al., 2019; Srivastava et al., 2019). Several studies support the regulatory mechanism of miRNAs in noncanonical TGF-β signaling pathway-mediated fibrotic diseases (Yang et al., 2016). Since miRNAs are commonly involved in regulating the canonical and noncanonical TGF-β signaling pathways, they have diagnostic and therapeutic potentials in many fibroproliferative diseases (Yang et al., 2016). The aim of this review is to comprehensively generalize the regulatory role of miRNAs in canonical and noncanonical TGF-β pathway-intervened fibrosis and identify their characteristics in different organs, with focus on the most recent literature to provide the current valuable knowledge in this area.

## Canonical TGF-β Signaling Pathway

In the canonical TGF-β signaling pathway, active TGF-β is first isolated from the latent TGF-β complex in the extracellular space, which maintains TGF-β in the inactive form (Meng et al., 2016). The inactive molecule then binds to the TGF-β type II receptor (TGF-βRII), which in turn binds and phosphorylates the TGF-β type I receptor ALK5, leading to its activation. Subsequently, Smad2 and Smad3 proteins are continuously phosphorylated by ALK5 in the cytoplasm and then form a complex with Smad4, which finally assembles in the nucleus to further regulate fibrosis-related transcription (Hill, 2009; Wrighton et al., 2009; Thatcher, 2010). In addition, the inhibitory function of SMAD-7 competes with SMAD-2/3 to bind with phosphorylated ALK5 (Yan et al., 2009; Hu et al., 2018).

## Noncanonical TGF-β Signaling Pathway

During fibrotic progression, complex regulatory networks composed of multiple TGF-β-related signaling pathways work together (Yang et al., 2016; Finnson et al., 2020). Therefore, in addition to canonical pathways, we should pay attention to noncanonical TGF-β signaling pathways, including ALK1/Smad1/5, Janus kinases (JAKs)/signal transducer and activator of transcription protein-3 (STAT3), phosphatidylinositol-3-kinase (PI3K), mitogen-activated protein (MAP) kinases (ERK, p38, and JNK), and rho-like GTPases in different cell types (Finnson et al., 2020).

Unlike the canonical ALK5-SMAD-2/3 pathway, the TGF-β type I receptor ALK1 forms a complex with ALK5 under stimulation by TGF-β, leading to activation of SMAD-1/5 and suppression of ALK5-SMAD-2/3 signaling (Goumans et al., 2003; Finnson et al., 2008). Moreover, the activation of SMAD-1 and SMAD-5 are both inhibited by SMAD-6 and SMAD-7, which respond to TGF-β-related bone morphogenetic proteins (Katsuno et al., 2018). Pannu et al. showed that in systemic sclerosis, the up-regulation of extracellular interstitial components, which depend on ALK5, is not associated with SMAD-2/3 activation but reconciled by ALK1/SMAD-1 and ERK-1/2 signalings (Pannu et al., 2007).

MAPK, which includes three major classical types, i.e., ERK, JNK, and p38 MAPK, is commonly involved in the up-regulation of key molecules that sustain cell proliferation, growth, differentiation, and survival processes (Braicu et al., 2019). After TGF-β-induced phosphorylation, TβRII and ALK5 can sequentially activate Ras, Raf, and MEK-1/2 to activate ERK-1/2 by recruiting adaptor protein growth factor receptor-bound protein 2/Son of Sevenless. Fibrosis-related genes, without or in collaboration with activated SMAD complexes, are controlled by some transcription factors, which are phosphorylated by activated ERK, such as jun and fos (Zhang, 2017). Moreover, ERKs can negatively regulate SMAD protein activity by directly phosphorylating the linker region of SMAD proteins (Kretzschmar et al., 1997; Kretzschmar et al., 1999). In fibrotic processes, intramolecular polyubiquitination of TNF receptor-associated factor 6 (TRAF-6) at Lys63 is conducted by the interplay between activated TGF-β receptors and TRAF-6 (Yamashita et al., 2008). Subsequently, TAK1 is recruited by polyubiquitinated TRAF-6 to activate JNK and p38 *via* activations of MKK4 and MKK3/6, respectively. Finally, the activated JNK and p38 continuously activate the downstream transcription factors c-jun and ATF-2, which then regulate SMAD activity through phosphorylation (Zhang, 2009; Zhang, 2017).

The PI3K/AKT/mammalian target of rapamycin (mTOR) signaling cascade, which comprises the core units of phosphatidylinositide 3-kinases and their downstream mediators AKT and mTOR, mediate cell proliferation, survival, and metabolism and control cellular differentiation (Yu and Cui, 2016; Marquard and Jücker, 2020). TGF-β regulates PI3K/AKT signaling through two mechanisms: SMAD-dependent and SMAD-independent (Zhang, 2017). In the SMAD-dependent pathway, TGF-β-driven SMAD signaling induces the lipid phosphatase SH2-containing inositol 5′-phosphatase, which can then inhibit PI3K/AKT signaling (Valderrama-Carvajal et al., 2002). Similar to TGF-β-induced JNK/p38 activation, in the SMAD-independent mechanism, ubiquitinated TRAF6 stimulates PI3K/AKT signaling and induces ubiquitination and activation of AKT (Yang et al., 2009). Although its specific function in fibrotic diseases is unclear, increasing evidence suggests that PI3K/AKT signaling plays a critical role in profibrotic processes in organs such as the liver, the heart, the kidney, the lungs, skin, and even oral mucosa (Dai et al., 2015; Li et al., 2017; Chaigne et al., 2019; Mi et al., 2019; Wang et al., 2019a; Zhou et al., 2019; Yin et al., 2020).

ROCK is the eventful cellular regulator of rho GTPases that organizes the actin cytoskeleton and facilitates myofibroblast differentiation and ECM production in fibroblasts of diverse tissues (Akhmetshina et al., 2008; Ji et al., 2014; Manickam et al., 2014; Lai et al., 2019). RhoA (a GTPase) and ROCK can be activated by TGF-β to induce actin polymerization through SMAD-dependent or SMAD-independent mechanisms(Zhang, 2017). The rho-like GTPases Cdc42 and Rac can also be recruited by TGF-β to combinate with the TGF-β receptor complex, which is involved in cell-to-cell connection dissociation and cell migration during epithelial to mesenchymal transition (EMT) (Zhang, 2017).

JAK, which is a receptor-associated tyrosine kinase, acts as regulators of cytokine and growth factor signaling. JAK2 is a regulator of the profibrotic results of TGF-β in many fibrotic diseases (O'Shea et al., 2015; Cao et al., 2019; Zhao et al., 2019; Qin et al., 2020). STAT3, a STAT protein and a downstream regulator of JAK2 signaling, is crucial to many fibrotic diseases in a TGF-β-dependent manner (O'Shea et al., 2015; Chakraborty et al., 2017; Oh et al., 2018). In this signaling, first, JAK2 is phosphorylated when cytokines bind to cell receptors. Subsequently, the phosphorylates downstream STAT3, which participates in various biological processes, such as cell proliferation, differentiation, and apoptosis (O'Shea et al., 2015).

## Role of miRNAs in the Canonical and Noncanonical TGF-β Signaling Pathways in Fibrotic Diseases Affecting Different Organs

### Kidney

In recent years, the vital role of miRNAs in renal pathophysiology has been evidenced by clinical and experimental models (Van der Hauwaert et al., 2019). During the progression of renal fibrosis mediated by SMAD-3, miR-29 and miR-200 are downregulated, and miR-21 and miR-192 are upregulated (Meng et al., 2015). The miR-29 family, including miR-29a, miR-29b, and miR-29c, acts as a negative modulator of some fibrotic diseases through canonical TGF-β-SMAD-2/3 signaling, thereby playing a protective role in the fibrotic process (He et al., 2013). Expression of disintegrin metalloproteases Adams, mainly Adam10, Adam12, Adam17, and Adam19, is significantly upregulated *in vivo* and *in vitro* through the regulation of the TGF-β-SMAD-2/3 pathway. Overexpression of miR-29 can downregulate Adam12 and Adam19 expressions, partially mitigating fibrosis (Ramdas et al., 2013). Although the function of Adam in fibrosis and its specific mechanism in the fibrotic process are clear, accumulating evidence indicates that they significantly correlate with the development of fibrosis and that multiple miR-29 binding sites are present in the 3′-UTR region of the mRNA of Adams (Ramdas et al., 2013). In addition, Sole et al. showed that patients with lupus nephritis accompanied by high renal chronicity indexes have significantly reduced miR-29c levels in urinary exosomes, suggesting the early diagnostic value of miR-29c in renal fibrosis (Solé et al., 2015). Although significant downregulation of miR-200b occurs in renal fibrosis related to TGF-β signaling (Meng et al., 2015). Tang et al. postulated that miR-200b represses TGF-β1-induced EMT by inhibiting ZEB1 and ZEB2 and ECM protein fibronectin by directly targeting the 3′UTR region of the mRNA, which is not directly associated with the TGF-β signaling pathway (Tang et al., 2013). A study showed that in paclitaxel-treated renal fibrosis animal models, inhibition of TGF-β/SMAD-2/3 signaling and mitigation of renal fibrosis, which were accompanied by downregulation of miR-192, probably implied the regulatory relationship between miR-192 and the canonical TGF-β signaling pathway (Sun et al., 2011). Diabetic nephropathy (DN) is characterized by basement membrane thickening, glomerular hypertrophy, and ECM deposition, eventually leading to renal interstitial fibrosis. Ma et al. showed that miR-130b is related to fibrosis in DN, and its overexpression can not only promote the mRNA and protein expressions of collagen types I and IV and fibronectin but also increase its downstream signal TGF-β1, t-Smad2/3, P‐SMAD-2/3, and SMAD-4 expressions, thus implying a profibrotic role of miR-130b in DN and its strong correlation with the canonical TGF-β/SMAD-2/3 signaling pathway (Ma et al., 2019). Another study verified the negative regulatory role of miR-101a in chronic kidney disease (Ding et al., 2020). KDM3A, a histone demethylase, which is a key regulator of histone modification, accelerates chronic renal fibrosis by directly regulating the YAP-TGF-β-SMAD signaling pathway or indirectly mediating TGF-β-SMAD signaling by suppressing the expression of TGIF1 (Ding et al., 2020). However, overexpressed miR-101a can alleviate chronic renal fibrosis by downregulating the protein and mRNA expressions of KDM3A. The inhibitory effect of miR-101a on this disease can also be partially reversed by overexpression of YAP/TGF-β2 or inhibition of TGIF (Ding et al., 2020). Taken together, the aforementioned miRNAs mainly affect the progression of renal fibrosis through the canonical TGF-β signaling pathway.

Several studies indicated that miR-21 is strongly associated with renal fibrosis and plays a key role in the profibrotic process (Zarjou et al., 2011; Ben-Dov et al., 2012; Glowacki et al., 2013; Chen et al., 2015; Luo et al., 2019). For example, in an *in vivo* study, the kidneys with unilateral ureteral obstruction presented with elevated levels of miR-21 compared to those without the obstruction in mouse models, (Glowacki et al., 2013) and blockade of miR-21 in this model alleviated fibrosis (Zarjou et al., 2011). In humans, the enhanced expression of miR-21 can be tested in renal allografts with severe interstitial fibrosis compared to those without fibrosis (Ben-Dov et al., 2012; Glowacki et al., 2013). A study by Chen et al. showed that elevated levels of miR-21 led to the upregulation of phosphorylated AKT and downregulation of PTEN, which sped up the fibrotic process during long-term nephrotoxicity induced by calcineurin inhibitors (Chen et al., 2015). In addition, Luo et al. indicated that Smilax glabra Roxb (PTFS), a traditional Chinese herb, showed powerful anti-EMT and anti-fibrosis effects both *in vitro* and *in vivo*, and the mechanism underlying them may be associated with the miR-21/PTEN/PI3K/Akt signaling pathway and their complicated upstream molecular regulatory network involving TGF-β signaling (Luo et al., 2019).

In early years, miR-132 was demonstrated to affect cell proliferation during wound healing by regulating the STAT3 and ERK pathways (Li et al., 2015a). Subsequently, Bijkerk et al. suggested that miR-132 may coordinately mediate genes involved in TGF-β signaling, STAT3/ERK pathways, and cell proliferation (Foxo3/p300) related to promoting trans-differentiation and proliferation of myofibroblasts during the formation of renal fibrosis (Bijkerk et al., 2016). A study by Wang et al. supported that p53 could physically interplay with the promoter region of miR-199a-3p by using chromatin immunoprecipitation assays (Wang et al., 2012a). Yang et al. reported a novel modulatory mechanism of promoting renal fibrosis in which miR-199a-3p suppresses the suppressors of cytokine signaling-7 (SOCS7), a SOCS family, to upregulate STAT3 activation, which is directly induced by TGF-β-driven p53 upregulation. *In vitro* and *in vivo* experiments also confirmed that the TGF-β/p53/miR-199a-3p/SOCS7/STAT3 axis may play a critical role in human renal fibrosis (Yang et al., 2017a). In addition to miR-199a-3p, miR-206 is associated with the JAK/STAT3 pathway, according to a study by Zhao et al. (2019). In that study, miR-206 attenuated EMT in chronic kidney disease by suppressing JAK/STAT signaling by directly targeting Annexin A1. Wu et al. reported that overexpression of miR-455-3p attenuates renal fibrosis by directly targeting the 3′-UTR region of ROCK2 in the DN model, providing a testimony for the protective effect of miR-455-3p in DN (Wu et al., 2018). Thus, these miRNAs affect renal fibrosis by regulating the non-canonical TGF-β signaling pathway.

### Liver

In studies related to liver fibrosis, miR-193a/b, miR-942, miR-96, and miR-21 play central roles in the canonical TGF-β signaling pathway (Roderburg et al., 2013; Luo et al., 2018; Tao et al., 2018; Ju et al., 2019). In concanavalin A‐induced hepatic fibrosis mice models, miR-193a/b-3p played a protective role by alleviating concanavalin A-induced hepatic fibrosis through apoptosis and cell cycle arrest of hepatic stellate cells (HSCs) and inhibition of HSCs activation (Ju et al., 2019). During this process, the expression of TGF-β1 and phosphorylation of SMAD-2/3 are restrained, and CAPRIN1, a cell cycle-related protein, is confirmed to be the target gene of miR-193a/b-3p by the dual luciferase reporter system (Ju et al., 2019). Another study showed the prominent role of miR-942 in the progression of liver fibrosis infected by the hepatitis B virus (Tao et al., 2018). The miR-942 in activated HSCs was upregulated in cell models and liver specimens of patients with hepatitis B virus-related liver fibrosis and correlated inversely with bone morphogenic proteins and activin membrane-bound inhibitor (BAMBI), which interfered with TGF-β1 signaling by capturing TGF-β receptor I (TβRI/ALK-5) (Tao et al., 2018). Thus, with the degradation of BAMBI, the activity of the TGF-β/SMAD-2/3 signaling pathway is enhanced, which promotes the process of fibrosis (Tao et al., 2018). Schistosomiasis, a severe subtropical parasitic disease, induces liver fibrosis and results in portal hypertension, which are the main causes of host mortality. Luo et al. (2018) indicated that schistosomiasis-infected mice showed significant hepatic fibrosis, accompanied by high expressions of miR-96, TGF-β, and Smad2/3 and suppression of Smad7 (Luo et al., 2018). Subsequently, transfection of the miR-96 inhibitor through recombinant adeno-associated virus 8 could significantly alleviate liver fibrosis, and the dual luciferase reporter assay proved that Smad7 is its target gene (Luo et al., 2018). A study indicated unchanged levels of overall miR-133a in whole RNA extracted from the fibrotic murine and human livers but specifically downregulated in HSC during fibrogenesis (Roderburg et al., 2013). The addition of TGF-β in HSC downregulated the expression of miR-133 more sharply, which aggravated the rate of hepatofibrogenesis, but overexpression of miR-133 could partly reverse this (Roderburg et al., 2013). Thus, miR-133 may be a diagnostic biomarker and a target for therapeutic strategies in hepatic fibrosis (Roderburg et al., 2013).

The role of miR-21 in liver fibrosis is supported by the literature (Yang et al., 2017b; Wang et al., 2017; Huang et al., 2019). In recent years, some studies have found that some drugs used for hepatic fibrosis exert anti-fibrosis effects by acting on miR-21-related signal axes (Yang et al., 2017b; Wang et al., 2017; Huang et al., 2019). The most reported drugs are chlorogenic acid (CGA) and methyl helicterate (Yang et al., 2017b; Wang et al., 2017; Huang et al., 2019). CGA, a phenolic acid abundantly found in nature, has various pharmacological effects, including anti-inflammatory, anti-hypertensive, and anti-oxidant capacities (Yang et al., 2017b). miR-21 can enhance the activity of the TGF-β/SMAD-2/3 signaling pathway by inhibiting the expression of Smad7, which promotes the fibrotic process in the liver, which is reversible by CGA *in vitro* and *in vivo* (Yang et al., 2017b). The therapeutic mechanism of CGA in liver fibrosis caused by schistosomiasis is also consistent with the aforementioned result, whereby significant downregulation of miR-21 expression is accompanied by upregulation of Smad7, reductions of p-Smad2 and p-Smad3, and conspicuous alleviation of fibrosis (Wang et al., 2017). Methyl helicterate is the main ingredient of *Helicteres angustifolia*, which has been utilized as a traditional Chinese medicine to treat immune disorders and liver diseases (Huang et al., 2019). Activation of the ERK1 pathway can promote the deposition of collagen and the expression of α-SMA to accelerate the process of fibrosis, while SPRY2 can inhibit this pathway, but miR-21 can bind to the 3′UTR region of SPRY2 to reverse its effect (Huang et al., 2019). A study showed that methyl helicterate could mitigate liver fibrosis by inhibiting miR-21-mediated ERK and TGFβ/SMAD 2/3 pathways and, therefore, has potential as a therapeutic drug for liver fibrosis (Huang et al., 2019).

Compared to normal liver tissue, miR-101 is significantly downregulated in hepatitis B virus-related cirrhosis, hepatic fibrosis, and hepatocellular carcinoma (Lei et al., 2019). A study showed that the expression of miR-101 in CCL_4_-induced fibrotic liver tissue in mice was significantly reduced, contrary to the high expressions of TGF-β, p-PI3K, p-Akt, p-mTOR, and fibrosis-related proteins (Lei et al., 2019). However, overexpression of miR-101 completely reversed this, so downregulating the PI3K/Akt/mTOR signaling pathway may be feasible against hepatic fibrosis (Lei et al., 2019). Similar to miR-101, miR-29b could also downregulate the PI3K/Akt signal axis to inhibit the fibrotic process in the liver (Wang et al., 2015). In addition, it could cause cell cycle arrest in the G1 phase of cells by downregulating cyclinD1 and p21, thereby suppressing hepatocyte viability and colony formation (Wang et al., 2015). In contrast, miR-33a plays a reverse role in promoting hepatic fibrosis by regulating the PI3K/Akt signaling pathway (Li et al., 2014). The activity of the JAK2/STAT3 signal axis in hepatic fibrosis also cannot be underestimated. Yang et al. reported that the low expression of miR-375-3p in the mouse liver can induce the JAK2/STAT3 pathway to activate the TGF-β1/SMAD signal and promote EMT, whereas the addition of miR-375-3p mimic has an antifibrotic effect (Yang et al., 2019). Taken together, these findings indicate the active regulatory functions of miRNAs in liver fibrosis affected by the non-canonical TGF-β signaling pathway.

### Lung

TGF-β receptors are important elements in the canonical TGF-β/SMAD 2/3 signaling pathway (Wrighton et al., 2009). Some studies reported that miRNAs, including miR-18a-5p, miR-153, and miR-1344, play anti-pulmonary fibrosis effects for targeting TGF-β receptors to inhibit downstream SMAD-2/3 expression (Liang et al., 2015; Stolzenburg et al., 2016; Zhang et al., 2017). Among them, miR-18a-5p and miR-153 directly target TGF-βRII, whereas miR-1344 can not only target TGF-βRII but also TGF-βRI (ALK5), which showed anti-fibrotic effects in lung fibrosis models (Liang et al., 2015; Stolzenburg et al., 2016; Zhang et al., 2017). miR-101 influences the proliferation and activation of lung fibroblasts in pulmonary fibrosis through two signaling pathways (Huang et al., 2017). Regarding the proliferation of pulmonary fibroblasts, miR-101 reverses this process by targeting frizzled receptor 4/6 (FZD4/6) to inhibit WNT5a-FZD4/6-NFATc2 signaling, which is a form of the Wnt signaling pathway (Huang et al., 2017). To activate lung fibroblasts, miR-101 reduces the degree of fibroblast activation by directly targeting TGF-βRI (ALK5) to further inhibit the phosphorylation of SMAD-2/3 (Huang et al., 2017). The expression of miR-411-3p in silicosis rats and lung fibroblasts induced by TGF-β was significantly reduced, and the overexpression of miR-411-3p reversed the phenomenon and relieved the development of pulmonary fibrosis (Gao et al., 2020). The underlying mechanism is that miR-411-3p has an inhibitory effect on the expression of SMAD ubiquitination regulator 2 (Smurf2) and reduces the ubiquitination degradation of Smad7 under control of Smurf2, which results in blocking TGF-β/Smads signaling (Gao et al., 2020). In contrast, miR-21 promotes the transduction of the TGF-β/SMAD-2/3 signaling pathway by directly targeting Smad7 to accelerate the process of pulmonary fibrosis (Wang et al., 2018). In addition, miR-21 can also sharply reduce the phosphorylation levels of ERK, p38, and JNK, which were induced by resveratrol, thereby promoting the process of fibrosis by affecting the non-canonical TGF-β/MAPK signaling pathway (Wang et al., 2018). The miR-200 family is useful in pulmonary fibrosis (Cao et al., 2018). An article reported that miR-200b/c exerts a protective effect by targeting ZEB1/2, which may be related to the inhibition of p38 MAPK and TGF-β/SMAD-3 signaling pathway (Cao et al., 2018). Autophagy, a method of fibrotic regulation, is significantly reduced during fibrosis, and a study showed that miR-449a activates autophagy by targeting Bcl2 induced by the TGF-β1/ERK/MAPK pathway, thereby alleviating the development of lung fibrosis (Han et al., 2016). In addition, miR-344a-5p plays a vital role in anti-pulmonary fibrosis through MAPK signaling pathways, including map3k11 (Liu et al., 2017). As for the mechanism, miR-344a-5p inhibits the proliferation of myofibroblasts by targeting the mRNA of map3k11 to alleviate pulmonary fibrosis (Liu et al., 2017). lncRNA PCF is upregulated, which is affected by the activation of TGF-β1, to act as the ceRNA of miR-344a-5p and reverse its effect (Liu et al., 2017).

In pulmonary fibrosis induced by the non-canonical TGF-β/PI3K-Akt signaling pathway, miR-193a, miR-542-5p, miR-31/184, and miR-301a are crucial (Yuan et al., 2018; Liu et al., 2019; Wang et al., 2020a). The upregulation of miR-193a in paraquat-induced pulmonary fibrosis leads to the downregulation of the PI3K/AKT/mTOR axis and the upregulation of its downstream autophagy-related LC3-II/LC3-I and Beclin1, which induced the autophagy of lung fibroblasts and then reduced the expression of pulmonary fibrosis marker protein α-SMA and collagen deposition (Liu et al., 2019). The decrease of miR-542-5p in silica-induced mouse pulmonary fibrosis was shown by the miRNAs microarray analysis in a study by Yuan et al. (2018) In that study, miR-542-5p was confirmed to reverse TGF-β1 or silica-induced mouse lung fibrosis by directly targeting integrin α6, which inhibited fibroblast activation and reduced the phosphorylation levels of FAK/PI3K/AKT *in vitro* (Yuan et al., 2018). MiR-31 and miR-184 play contradictory roles in pulmonary fibrosis, which was verified by Wang et al. (2020a). In that study, miR-31 was enhanced, and miR-184 was suppressed *in vitro*, accompanied by the upregulation of TGF-β-SMAD-2 and TGF-β-PI3K-AKT signaling pathways and increase of some profibrotic factors, matrix metallopeptidase 7 (MMP7) and Runt-related protein 2 (RUNX2) (Wang et al., 2020a). As for the mechanism, the 3′UTR region of SMAD 6 was confirmed to be the binding site of miR-31, which promoted SMAD 2 phosphorylation to further enhance the SMAD 2/SMAD-4 dimer formation and translocation (Wang et al., 2020a). Thus, downregulating miR-31 and upregulating miR-184 may effectively ameliorate pulmonary fibrosis (Wang et al., 2020a). In the noncanonical TGF-β/JAK2/STAT3 pathway in lung fibrosis, miR-125a-3p plays a pivotal role. In a study by Xu et al., miR-125a-3p directly targetted 3′UTR of Fyn and then lead to the inactivation of the Fyn downstream effector STAT3, which inhibited the progression of silica-induced murine pulmonary fibrosis and TGF-β1-treated fibroblast lines (Xu et al., 2019).

### Heart

In cardiac fibrosis, miRNAs related to the regulation of the canonical TGF-β/Smad2/3 signaling pathway should first be miR-150-5p (Che et al., 2019). In a study by Che et al., miR-150-5p inhibited the binding of Smad7 to ALK5 by directly targeting smad7, which promoted the increase of the binding of Smad2/3 to ALK5, thereby promoting the high expression of TGF-β/Smad2/3 signal, accelerating extracellular collagen deposition, and exacerbating the process of myocardial fibrosis (Che et al., 2019). miR-328 has the same effect as miR-150-5p in cardiac fibrosis, and its inhibition can also significantly reduce the expression level of the TGF-β/Smad2/3 signaling pathway, but its specific target molecule is unclear (Du et al., 2016). The canonical effectors of the TGF-β signaling pathway, p-SMAD2 and p-SMAD3, can also control the nuclear steps of miRNA biogenesis, which promotes transcriptional activation of pri-miRNAs and regulates the subsequent post-transcriptional conversion of pri-miRNAs into pre-miRNAs by DROSHA (Siomi and Siomi, 2010). This mechanism is well reflected in the regulation of the conversion of pri-miR-21 into pre-miR-21 (Davis-Dusenbery and Hata, 2011). Moreover, p-SMAD2/3 can interact with the DICER enzyme in the cytoplasm to enhance the cleavage efficiency of DICER for pre-miR-21 in the form of a complex of p-SMAD2/3/DICER, which promotes pre-miR-21 to mature miR-21 transformation (García et al., 2015). In addition, the direct target effect of miR-21 on the 3′UTR region of SMAD7 in cardiac fibrosis was demonstrated in that article, which further clarified the pro-fibrotic mechanism of miR-21 (García et al., 2015). Unlike the aforementioned miRNAs, miR-24 has the contradictory effect of inhibiting cardiac fibrosis (Wang et al., 2012b). The overexpressed miR-24 downregulates the expression of the TGF-β/SMAD2/3 signaling pathway to slow down the fibrosis process by directly targeting furin, which is a protease that controls latent TGF-β activation processing (Wang et al., 2012b).

In the non-canonical TGF-β/MAPKs signaling pathway, a study on miR-433 characterized its crucial role in regulating cardiac fibrosis (Tao et al., 2016). In that study, miR-433 was highly expressed in fibroblasts of cardiac fibrosis tissue, and its knockdown could significantly inhibit the transdifferentiation of cardiac fibroblasts into myofibroblasts (Tao et al., 2016). As for the mechanism, miR-433 directly targets JNK1, which leads to the activation of ERK and p38 kinase, and subsequent SMADs activity, particularly SMAD3 activity (Tao et al., 2016). miR-7a/b has a contradictory effect compared to miR-433 in the progression of cardiac fibrosis (Li et al., 2015b). Although the potential target of miR-7a/b is unclear, it similarly affects TGF-β/MAPKs signaling in the dynamic of the regulation of cardiac fibrosis (Li et al., 2015b).

### Other Organs

Hypertrophic scar (HS) is a pathological scar resulting from abnormal wound healing (Shen et al., 2020). A study by Shen et al. reported that miR-145-5p could remarkably ameliorate HS by directly targeting the canonical TGF-β effectors SMAD-2/3 (Shen et al., 2020). Thus, it may be an available therapeutic drug for treating HS (Shen et al., 2020). The same effect could be found in the regulated function of miR-29b in HS, but its specific target in the canonical TGF-β signaling pathway requires further research (Guo et al., 2017).

Bladder outlet obstruction (BOO) is commonly encountered in the field of urology, often accompanying fibrosis of the bladder structure (Fusco et al., 2018). Wang et al. showed that miR-101b has protective function in hypoxia-induced fibrosis coursed by BOO, which is mainly associated with the canonical TGF-β/SMAD-2/3 signaling pathway (Wang et al., 2019b). As for the mechanism, the TGF-β type 1 receptor was inhibited by direct targeting of miR-101b, subsequently affecting the phosphorylation of its downstream molecule SMAD2/3, which ultimately caused the downregulation of the canonical TGF-β/SMAD2/3 signaling pathway (Wang et al., 2019b).

**FIGURE 1:**
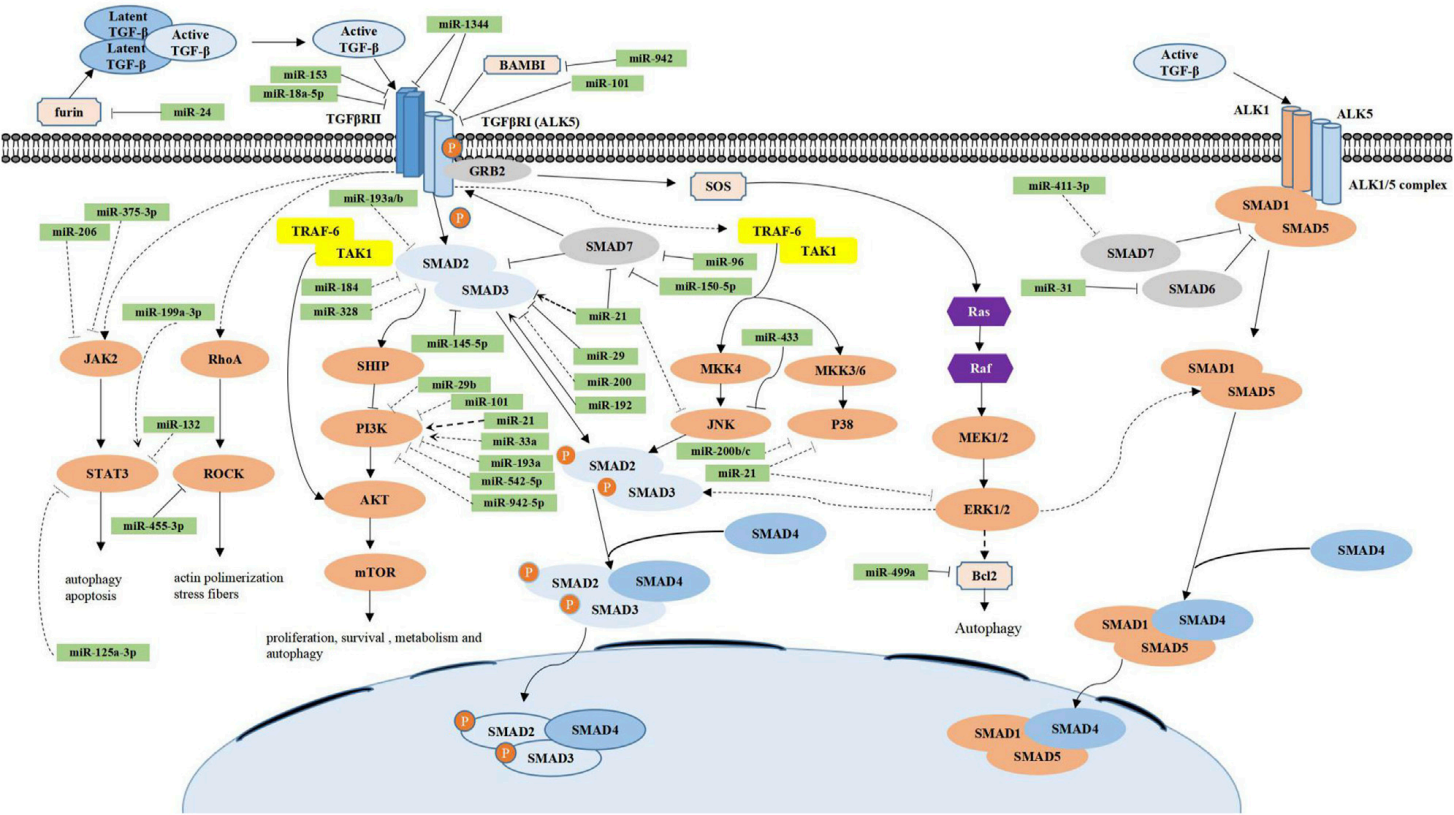
Regulatory mechanisms of miRNAs in the canonical and noncanonical TGF-β signaling pathways. In the canonical TGF-β signaling pathway (blue), active TGF-β is first isolated from the latent TGF-β complex in the extracellular space and then binds to the TGF-β type II receptor (TGF-βRII), which in turn binds and phosphorylates TGF-β type I receptor (ALK5), causing its activation. ALK5 continues to phosphorylate intracellular SMAD-2 and SMAD-3 proteins, which constitute a complex with SMAD-4 and finally accumulate in the nucleus and further regulate fibrosis-related gene expression. In addition, the inhibitory function of SMAD-7 competes with SMAD 2/3 to bind with phosphorylated ALK5, thereby inhibiting the TGF-β signaling pathway. The noncanonical TGF-β signaling pathway (orange) most commonly includes the TGF-β/Smad1/5, TGF-β/PI3K/AKT/mTOR, TGF-β/MAPK (ERKs, JNKs, and p38), TGF-β/JAK2/STAT3, and TGF-β/RhoA/ROCK signaling pathways. Their functions are also listed in the figure. Numerous miRs have been implicated in the regulation of the canonical and noncanonical TGF-β signaling pathways. miRs are grouped to have the direct (solid line) or indirect (dotted line) effect as well as the promotion (arrowhead) or inhibition (flathead) effect.

Oral submucous fibrosis (OSMF) is a chronic, progressive, pre-malignant condition mainly related to the consumption of betel nuts (Singh et al., 2018). In recent years, many studies have investigated miRNAs related to OSMF, including miR-21, miR-10b, and miR-942-5p (Singh et al., 2018; Fang et al., 2020; Wang et al., 2020b). Among them, the most in-depth research is of miR-942-5p, reported by Wang et al. (2020b) In that study, miR-942-5p showed significantly low expression in oral squamous carcinoma in the background of OSMF and could directly target latent transforming growth factor beta binding protein 2 (LTBP2), which further blocked the PI3K/Akt/mTOR signaling pathway (Wang et al., 2020b). The regulatory mechanisms of miRNAs in the canonical and non-canonical TGF-β signaling pathway are summarized as shown in Figure 1. In addition, the miRNAs in canonical and non-canonical TGF-β signaling in fibrotic diseases of different organs are also listed in the Table 1.

**TABLE 1:** MiRNAs in canonical and noncanonical TGF-β signaling in fibrotic diseases of different organs. HS, hypertrophic scar; BOO, bladder outlet obstruction; OSF, oral submucous fibrosis; TGF-β, transforming growth factor-β; Smad2/3, *drosophila* mothers against decapentaplegic protein 2/3; PI3K, phosphatidylinositol 3-kinase; Akt, protein kinase B; MAPK, mitogen-activated protein kinase; JAK2, Janus kinase 2; STAT3, signal transducer and activator of transcription 3; ROCK, rho-associated coiled-coil containing kinase.

Location	Source	Disease	Mechanism	microRNAs	References
Kidney	Human	Renal fibrosis	TGF-β/Smad2/3 signaling pathway	miR-29	He et al. (2013), Ramdas et al. (2013), Solé et al. (2015)
			TGF-β/Smad2/3 signaling pathway	miR-21	Glowacki et al. (2013), Zarjou et al. (2011), Ben-Dov et al. (2012)
			TGF-β/Smad2/3 signaling pathway	miR-192	Sun et al. (2011)
			TGF-β/Smad2/3 signaling pathway	miR-101a	Ding et al. (2020)
			TGF-β/PI3K/Akt signaling pathway	miR-21	Chen et al. (2015), Luo et al. (2019)
			TGF-β/JAK2/STAT3 signaling pathway	miR-132	Li et al. (2015a), Bijkerk et al. (2016)
			TGF-β/JAK2/STAT3 signaling pathway	miR-199a-3p	Wang et al. (2012a), Yang et al. (2017a)
			TGF-β/JAK2/STAT3 signaling pathway	miR-206	Zhao et al. (2019)
			TGF-β/ROCK signaling pathway	miR-455-3p	Wu et al. (2018)
Liver	Human	Hepatic fibrosis	TGF-β/Smad2/3 signaling pathway	miR-193a/b	Ju et al. (2019)
			TGF-β/Smad2/3 signaling pathway	miR-942	Tao et al. (2018)
			TGF-β/Smad2/3 signaling pathway	miR-96	Luo et al. (2018)
			TGF-β/Smad2/3 signaling pathway	miR-133	Roderburg et al. (2013)
			TGF-β/Smad2/3 signaling pathway	miR-21	Yang et al. (2017b), Wang et al. (2017), Huang et al. (2019)
			TGF-β/PI3K/Akt signaling pathway	miR-101	Lei et al. (2019)
			TGF-β/PI3K/Akt signaling pathway	miR-29b	Wang et al. (2015)
			TGF-β/PI3K/Akt signaling pathway	miR-33a	Li et al. (2014)
			TGF-β/JAK2/STAT3 signaling pathway	miR-375-3p	Yang et al. (2019)
			TGF-β/MAPK signaling pathway	miR-21	Huang et al. (2019)
Lung	Human	Pulmonary fibrosis	TGF-β/Smad2/3 signaling pathway	miR-18a-5p	Zhang et al. (2017)
			TGF-β/Smad2/3 signaling pathway	miR-153	Liang et al. (2015)
			TGF-β/Smad2/3 signaling pathway	miR-1344	Stolzenburg et al. (2016)
			TGF-β/Smad2/3 signaling pathway	miR-411-3p	Gao et al. (2020)
			TGF-β/Smad2/3 signaling pathway	miR-21	Wang et al. (2018)
			TGF-β/Smad2/3 signaling pathway	miR-200b/c	Cao et al. (2018)
			TGF-β/MAPK signaling pathway	miR-21	Wang et al. (2018)
			TGF-β/MAPK signaling pathway	miR-344a-5p	Liu et al. (2017)
			TGF-β/MAPK signaling pathway	miR-200b/c	Cao et al. (2018)
			TGF-β/PI3K/Akt signaling pathway	miR-193a	Liu et al. (2019)
			TGF-β/PI3K/Akt signaling pathway	miR-542-5p	Yuan et al. (2018)
			TGF-β/PI3K/Akt signaling pathway	miR-31/184	Wang et al. (2020a)
			TGF-β/JAK2/STAT3 signaling pathway	miR-125a-3p	Xu et al. (2019)
Heart	Human	Cardiac fibrosis	TGF-β/Smad2/3 signaling pathway	miR-150-5p	Che et al. (2019)
			TGF-β/Smad2/3 signaling pathway	miR-328	Du et al. (2016)
			TGF-β/Smad2/3 signaling pathway	miR-21	Davis-Dusenbery and Hata (2011), García et al. (2015)
			TGF-β/Smad2/3 signaling pathway	miR-24	Wang et al. (2012b)
			TGF-β/MAPK signaling pathway	miR-433	Tao et al. (2016)
Other organs	Human	HS	TGF-β/Smad2/3 signaling pathway	miR-145-5p	Shen et al. (2020)
			TGF-β/Smad2/3 signaling pathway	miR-29b	Guo et al. (2017)
		BOO	TGF-β/Smad2/3 signaling pathway	miR-101b	Fusco et al. (2018)
		OSF	TGF-β/PI3K/Akt signaling pathway	miR-942-5p	Wang et al. (2020b)
			TGF-β/PI3K/Akt signaling pathway	miR-21	Singh et al. (2018)

## Discussion

Previous studies have suggested that the TGF-β signaling pathway, which plays a major regulatory role in fibrotic diseases, is widely regulated by miRNAs. Some of these miRNAs can affect various fibrotic diseases *via* canonical or non-canonical TGF-β signaling, including miR-21, miR-101, miR-29, miR-200, miR-942, and miR-193. Among them, miR-21 has been widely confirmed to promote the occurrence and development of fibrosis of organs, such as the kidney (Zarjou et al., 2011; Ben-Dov et al., 2012; Glowacki et al., 2013; Chen et al., 2015; Luo et al., 2019), liver (Yang et al., 2017b; Wang et al., 2017; Huang et al., 2019), lung (Wang et al., 2018) and heart (Davis-Dusenbery and Hata, 2011; García et al., 2015), by targeting the canonical TGF-β/SMAD2/3 signaling pathway and the non-canonical TGF-β/PI3K/AKT signaling pathway. miR-101 plays an active role in inhibiting fibrotic diseases, such as renal (Ding et al., 2020), hepatic (Lei et al., 2019), or pulmonary fibrosis (Huang et al., 2017) and bladder outlet obstruction (Wang et al., 2019b), by directly targeting ALK5 in the canonical TGF-β signaling pathway. miR-29 can ameliorate renal fibrosis (Ramdas et al., 2013; Solé et al., 2015) and HS (Guo et al., 2017) by targeting the canonical TGF-β signaling-related disintegrin metalloprotease Adams but inhibit liver fibrosis (Wang et al., 2015) by regulating the non-canonical TGF-β/PI3K/AKT signaling pathway. miR-200 exerts anti-fibrosis effects on the kidney (Tang et al., 2013) and lung (Cao et al., 2018) by directly targeting ZEB1/2. miR-942 can promote liver fibrosis (Tao et al., 2018) but plays a contrary regulatory role in oral submucous fibrosis (Wang et al., 2020b). In addition, miR-193 plays a protective role in pulmonary (Liu et al., 2019) and hepatic fibrosis (Ju et al., 2019) by regulating canonical TGF-β/SMAD2/3 and non-canonical TGF-β/PI3K/AKT/mTOR signaling pathways, respectively. Thus, the aforementioned miRNAs can become therapeutic targets or molecular drugs for fibrotic diseases owing to their regulatory effects in various fibrotic diseases.

Fibrosis offers the advantage of promoting tissue repair and healing. However, excessive fibrosis can cause tissue and organ dysfunctions (Diegelmann and Evans, 2004). During tissue repair, temporary ECM deposition occurs first, followed by the recruited inflammatory cells, the proliferated fibroblasts, and angiogenesis replacing the temporary ECM deposition and, finally, capillaries and fibroblasts degenerating with epithelial regeneration (Diegelmann and Evans, 2004). In contrast, fibrosis represents a disorder of the tissue repair process, during which excessive deposition of ECM rich in fibrous collagen, induction and proliferation of myofibroblasts, and repeated inflammatory responses lead to the replacement of normal parenchymal tissues and formation of non-functioning scar tissue (Diegelmann and Evans, 2004). Canonical and non-canonical TGF-β signaling pathways are the main signal axis regulating the fibrosis process (Meng et al., 2016; Finnson et al., 2020). miRNAs can promote or inhibit the progression of fibrotic diseases by targeting the upstream or downstream signal molecules of the TGF-β signaling network (Banerjee et al., 2011; Liu et al., 2018; Li et al., 2019). Therefore, miRNAs can be used as molecular drugs or targets to diagnose and treat fibrotic diseases. The intervention of miRNAs in the early manifestations of fibrosis related to the TGF-β signaling pathway may induce tissue healing and prevent the occurrence of fibrosis.

## Conclusion and Prospects

Fibrosis is often the final histopathological change in the development of chronic inflammatory diseases and can occur in almost all tissues and organs throughout the body. According to the current research results of fibrotic diseases of various organs, the developmental mechanisms of tissue and organ fibrosis mainly involve the canonical TGF-β/SMAD2/3 signaling pathway and the non-canonical TGF-β pathway, including the TGF-β/ALK1/Smad1/5 pathway, the TGF-β/MAPK pathway, the TGF-β/PI3K-Akt pathway, the TGF-β/JAK2/STAT3 pathway, and the TGF-β/ROCK pathway. MiRNA, which is involved in various physiological and pathological processes, with important roles in the canonical or noncanonical TGF-β signaling pathways, has received widespread attention in recent years. The regulating function of miRNAs in fibrosis is mainly to promote or ameliorate fibrosis by inhibiting the expression of effect molecules in the fibrosis signal pathway at the mRNA level. However, current research on the regulation of miRNA in fibrotic diseases mainly focuses on fibrosis of organs such as the liver, the kidneys, the lungs, and the heart, whereas the fibrosis-related research of other tissues and organs is limited and not in-depth. In addition, for the current mechanism research, the specific targets of many miRNAs and the upstream and downstream regulatory relationships are not comprehensive or sufficiently thorough.

In the future, many questions concerning the mechanisms of the pathogenesis of fibrotic diseases should be solved. For example, in addition to the few aforementioned organs, fibrosis of other tissues and organs has unique features and developmental progression, carrying research value. Therefore, more in-depth research should be performed. There may be more than five non-canonical TGF-β signaling pathways that affect the development of fibrosis. The related miRNAs are not limited to pathways, and long-term continuous exploration is required. The pathogenesis of fibrotic diseases is complex. Therefore, a single target therapy cannot have a complete effect, and combination treatment with multiple targets and signaling pathway may be more reasonable. Hence, the next step should be to explore the mechanisms of fibrotic diseases from different tissue and organs, investigating more signaling pathways related to fibrosis and more functional miRNAs in these diseases and trying combination therapeutic methods with multiple targets and multiple pathways.

## References

[B1] Akhmetshina A. Dees C. Pileckyte M. Szucs G. Spriewald B. M. Zwerina J. (2008). Rho-associated Kinases Are Crucial for Myofibroblast Differentiation and Production of Extracellular Matrix in Scleroderma Fibroblasts. Arthritis Rheum. 58, 2553–2564. 10.1002/art.23677 18668558

[B2] Banerjee J. Chan Y. C. Sen C. K. (2011). MicroRNAs in Skin and Wound Healing. Physiol. Genomics 43, 543–556. 10.1152/physiolgenomics.00157.2010 20959495 PMC3110888

[B3] Ben-Dov I. Z. Muthukumar T. Morozov P. Mueller F. B. Tuschl T. Suthanthiran M. (2012). MicroRNA Sequence Profiles of Human Kidney Allografts with or without Tubulointerstitial Fibrosis. Transplantation 94, 1086–1094. 10.1097/TP.0b013e3182751efd 23131772 PMC3541003

[B4] Bijkerk R. de Bruin R. G. van Solingen C. van Gils J. M. Duijs J. M. G. J. van der Veer E. P. (2016). Silencing of microRNA-132 Reduces Renal Fibrosis by Selectively Inhibiting Myofibroblast Proliferation. Kidney Int. 89, 1268–1280. 10.1016/j.kint.2016.01.029 27165825

[B5] Braicu C. Buse M. Busuioc C. Drula R. Gulei D. Raduly L. (2019). A Comprehensive Review on MAPK: A Promising Therapeutic Target in Cancer. Cancers 11, 1618. 10.3390/cancers11101618 PMC6827047 31652660

[B6] Cao G. Zhu R. Jiang T. Tang D. Kwan H. Y. Su T. (2019). Danshensu, a Novel Indoleamine 2,3-dioxygenase1 Inhibitor, Exerts Anti-hepatic Fibrosis Effects via Inhibition of JAK2-STAT3 Signaling. Phytomedicine 63, 153055. 10.1016/j.phymed.2019.153055 31377585

[B7] Cao Y. Liu Y. Ping F. Yi L. Zeng Z. Li Y. (2018). miR-200b/c Attenuates Lipopolysaccharide-Induced Early Pulmonary Fibrosis by Targeting ZEB1/2 via P38 MAPK and TGF-β/smad3 Signaling Pathways. Lab. Invest. 98, 339–359. 10.1038/labinvest.2017.123 29200203

[B8] Chaigne B. Clary G. Le Gall M. Dumoitier N. Fernandez C. Lofek S. (2019). Proteomic Analysis of Human Scleroderma Fibroblasts Response to Transforming Growth Factor‐ß. Prot. Clin. Appl. 13, 1800069. 10.1002/prca.201800069 30141531

[B9] Chakraborty D. Šumová B. Mallano T. Chen C.-W. Distler A. Bergmann C. (2017). Activation of STAT3 Integrates Common Profibrotic Pathways to Promote Fibroblast Activation and Tissue Fibrosis. Nat. Commun. 8, 1130. 10.1038/s41467-017-01236-6 29066712 PMC5654983

[B10] Che H. Wang Y. Li Y. Lv J. Li H. Liu Y. (2019). Inhibition of microRNA‐150‐5p Alleviates Cardiac Inflammation and Fibrosis via Targeting Smad7 in High Glucose‐treated Cardiac Fibroblasts. J. Cel Physiol 235, 7769–7779. 10.1002/jcp.29386 31710102

[B11] Chen J. Zmijewska A. Zhi D. Mannon R. B. (2015). Cyclosporine-mediated Allograft Fibrosis Is Associated with Micro-RNA-21 through AKT Signaling. Transpl. Int. 28, 232–245. 10.1111/tri.12471 25266172

[B12] Dai J.-P. Zhu D.-X. Sheng J.-T. Chen X.-X. Li W.-Z. Wang G.-F. (2015). Inhibition of Tanshinone IIA, Salvianolic Acid A and Salvianolic Acid B on Areca Nut Extract-Induced Oral Submucous Fibrosis *In Vitro* . Molecules 20, 6794–6807. 10.3390/molecules20046794 25884554 PMC6272768

[B13] Davis-Dusenbery B. N. Hata A. (2011). Smad-mediated miRNA Processing. RNA Biol. 8, 71–76. 10.4161/rna.8.1.14299 21289485 PMC3230544

[B14] Diegelmann R. F. Evans M. C. (2004). Wound Healing: An Overview of Acute, Fibrotic and Delayed Healing. Front. Biosci. 9, 283–289. 10.2741/1184 14766366

[B15] Ding H. Xu Y. Jiang N. (2020). Upregulation of miR-101a Suppresses Chronic Renal Fibrosis by Regulating KDM3A via Blockade of the YAP-TGF-β-Smad Signaling Pathway. Mol. Ther. - Nucleic Acids 19, 1276–1289. 10.1016/j.omtn.2020.01.002 32092824 PMC7033461

[B16] Du W. Liang H. Gao X. Li X. Zhang Y. Pan Z. (2016). MicroRNA-328, a Potential Anti-fibrotic Target in Cardiac Interstitial Fibrosis. Cell Physiol Biochem 39, 827–836. 10.1159/000447793 27497782

[B17] Fang C.-Y. Yu C.-C. Liao Y.-W. Hsieh P.-L. Ohiro Y. Chu P.-M. (2020). miR-10b Regulated by Twist Maintains Myofibroblasts Activities in Oral Submucous Fibrosis. J. Formos. Med. Assoc. 119, 1167–1173. 10.1016/j.jfma.2020.03.005 32265096

[B18] Finnson K. W. Almadani Y. Philip A. (2020). Non-canonical (Non-smad2/3) TGF-β Signaling in Fibrosis: Mechanisms and Targets. Semin. Cel Develop. Biol. 101, 115–122. 10.1016/j.semcdb.2019.11.013 31883994

[B19] Finnson K. W. Parker W. L. ten Dijke P. Thorikay M. Philip A. (2008). ALK1 Opposes ALK5/Smad3 Signaling and Expression of Extracellular Matrix Components in Human Chondrocytes. J. Bone Miner Res. 23, 896–906. 10.1359/jbmr.080209 18333754

[B20] Fusco F. Creta M. De Nunzio C. Iacovelli V. Mangiapia F. Li Marzi V. (2018). Progressive Bladder Remodeling Due to Bladder Outlet Obstruction: a Systematic Review of Morphological and Molecular Evidences in Humans. Bmc Urol. 18, 15. 10.1186/s12894-018-0329-4 29519236 PMC5844070

[B21] Gao X. Xu H. Xu D. Li S. Wei Z. Li S. (2020). MiR-411-3p Alleviates Silica-Induced Pulmonary Fibrosis by Regulating Smurf2/TGF-β Signaling. Exp. Cel Res. 388, 111878. 10.1016/j.yexcr.2020.111878 32004504

[B22] García R. Nistal J. F. Merino D. Price N. L. Fernández-Hernando C. Beaumont J. (2015). p-SMAD2/3 and DICER Promote Pre-miR-21 Processing during Pressure Overload-Associated Myocardial Remodeling. Biochim. Biophys. Acta (Bba) - Mol. Basis Dis. 1852, 1520–1530. 10.1016/j.bbadis.2015.04.006 25887159

[B23] Glowacki F. Savary G. Gnemmi V. Buob D. Van der Hauwaert C. Lo-Guidice J.-M. (2013). Increased Circulating miR-21 Levels Are Associated with Kidney Fibrosis. PLoS One 8, e58014. 10.1371/journal.pone.0058014 23469132 PMC3585177

[B24] Goumans M.-J. Valdimarsdottir G. Itoh S. Lebrin F. Larsson J. Mummery C. (2003). Activin Receptor-like Kinase (ALK)1 Is an Antagonistic Mediator of Lateral TGFβ/ALK5 Signaling. Mol. Cel 12, 817–828. 10.1016/s1097-2765(03)00386-1 14580334

[B25] Guo J. Lin Q. Shao Y. Rong L. Zhang D. (2017). miR-29b Promotes Skin Wound Healing and Reduces Excessive Scar Formation by Inhibition of the TGF-β1/Smad/CTGF Signaling Pathway. Can. J. Physiol. Pharmacol. 95, 437–442. 10.1139/cjpp-2016-0248 28092445

[B26] Han R. Ji X. Rong R. Li Y. Yao W. Yuan J. (2016). MiR-449a Regulates Autophagy to Inhibit Silica-Induced Pulmonary Fibrosis through Targeting Bcl2. J. Mol. Med. 94, 1267–1279. 10.1007/s00109-016-1441-0 27351886

[B27] He Y. Huang C. Lin X. Li J. (2013). MicroRNA-29 Family, a Crucial Therapeutic Target for Fibrosis Diseases. Biochimie 95, 1355–1359. 10.1016/j.biochi.2013.03.010 23542596

[B28] Henry T. W. Mendoza F. A. Jimenez S. A. (2019). Role of microRNA in the Pathogenesis of Systemic Sclerosis Tissue Fibrosis and Vasculopathy. Autoimmun. Rev. 18, 102396. 10.1016/j.autrev.2019.102396 31520794

[B29] Hill C. S. (2009). Nucleocytoplasmic Shuttling of Smad Proteins. Cell Res 19, 36–46. 10.1038/cr.2008.325 19114992

[B30] Hu H.-H. Chen D.-Q. Wang Y.-N. Feng Y.-L. Cao G. Vaziri N. D. (2018). New Insights into TGF-β/Smad Signaling in Tissue Fibrosis. Chemico-Biological Interactions 292, 76–83. 10.1016/j.cbi.2018.07.008 30017632

[B31] Huang C. Xiao X. Yang Y. Mishra A. Liang Y. Zeng X. (2017). MicroRNA-101 Attenuates Pulmonary Fibrosis by Inhibiting Fibroblast Proliferation and Activation. J. Biol. Chem. 292, 16420–16439. 10.1074/jbc.M117.805747 28726637 PMC5633105

[B32] Huang Q. Zhang X. Bai F. Nie J. Wen S. Wei Y. (2019). Methyl Helicterte Ameliorates Liver Fibrosis by Regulating miR-21-Mediated ERK and TGF-β1/Smads Pathways. Int. Immunopharmacology 66, 41–51. 10.1016/j.intimp.2018.11.006 30419452

[B33] Ikushima H. Miyazono K. (2012). TGF-β Signal Transduction Spreading to a Wider Field: a Broad Variety of Mechanisms for Context-dependent Effects of TGF-β. Cell Tissue Res 347, 37–49. 10.1007/s00441-011-1179-5 21618142

[B34] Ji H. Tang H. Lin H. Mao J. Gao L. Liu J. (2014). Rho/Rock Cross-Talks with Transforming Growth Factor-β/Smad Pathway Participates in Lung Fibroblast-Myofibroblast Differentiation. Biomed. Rep. 2, 787–792. 10.3892/br.2014.323 25279146 PMC4179758

[B35] Ju B. Nie Y. Yang X. Wang X. Li F. Wang M. (2019). miR‐193a/b‐3p Relieves Hepatic Fibrosis and Restrains Proliferation and Activation of Hepatic Stellate Cells. J. Cel Mol Med 23, 3824–3832. 10.1111/jcmm.14210 PMC6533489 30945448

[B36] Katsuno Y. Qin J. Oses-Prieto J. Wang H. Jackson-Weaver O. Zhang T. (2018). Arginine Methylation of SMAD7 by PRMT1 in TGF-β-Induced Epithelial-Mesenchymal Transition and Epithelial Stem-Cell Generation. J. Biol. Chem. 293, 13059–13072. 10.1074/jbc.RA118.002027 29907569 PMC6109915

[B37] Kretzschmar M. Doody J. Massagu J. (1997). Opposing BMP and EGF Signalling Pathways Converge on the TGF-β Family Mediator Smad1. Nature 389, 618–622. 10.1038/39348 9335504

[B38] Kretzschmar M. Doody J. Timokhina I. Massague J. (1999). A Mechanism of Repression of TGFbeta/Smad Signaling by Oncogenic Ras. Genes Develop. 13, 804–816. 10.1101/gad.13.7.804 10197981 PMC316599

[B39] Lai S. S. Fu X. Cheng Q. Yu Z. H. Jiang E. Z. Zhao D. D. (2019). HSC-specific Knockdown of GGPPS Alleviated CCl4-Induced Chronic Liver Fibrosis through Mediating RhoA/Rock Pathway. Am. J. Transl Res. 11, 2382–2392. 31105844 PMC6511779

[B40] Lei Y. Wang Q.-I. Shen L. Tao Y.-Y. Liu C.-H. (2019). MicroRNA-101 Suppresses Liver Fibrosis by Downregulating PI3K/Akt/mTOR Signaling Pathway. Clin. Res. Hepatol. Gastroenterol. 43, 575–584. 10.1016/j.clinre.2019.02.003 30857885

[B41] Li D. Wang A. Liu X. Meisgen F. Grünler J. Botusan I. R. (2015a). MicroRNA-132 Enhances Transition from Inflammation to Proliferation during Wound Healing. J. Clin. Invest. 125, 3008–3026. 10.1172/jci79052 26121747 PMC4563743

[B42] Li K. Wu Y. Yang H. Hong P. Fang X. Hu Y. (2019). H19/miR‐30a/C8orf4 axis Modulates the Adipogenic Differentiation Process in Human Adipose Tissue‐derived Mesenchymal Stem Cells. J. Cel Physiol 234, 20925–20934. 10.1002/jcp.28697 31026067

[B43] Li R. Xiao J. Qing X. Xing J. Xia Y. Qi J. (2015b). Sp1 Mediates a Therapeutic Role of MiR-7a/b in Angiotensin II-Induced Cardiac Fibrosis via Mechanism Involving the TGF-β and MAPKs Pathways in Cardiac Fibroblasts. Plos One 10, e0125513. 10.1371/journal.pone.0125513 25923922 PMC4414609

[B44] Li X. Zhang Z.-L. Wang H.-F. (2017). Fusaric Acid (FA) Protects Heart Failure Induced by Isoproterenol (ISP) in Mice through Fibrosis Prevention via TGF-β1/SMADs and PI3K/AKT Signaling Pathways. Biomed. Pharmacother. 93, 130–145. 10.1016/j.biopha.2017.06.002 28624424

[B45] Li Z.-J. Ou-Yang P.-H. Han X.-P. (2014). Profibrotic Effect of miR-33a with Akt Activation in Hepatic Stellate Cells. Cell Signal. 26, 141–148. 10.1016/j.cellsig.2013.09.018 24100264

[B46] Liang C. Li X. Zhang L. Cui D. Quan X. Yang W. (2015). The Anti-fibrotic Effects of microRNA-153 by Targeting TGFBR-2 in Pulmonary Fibrosis. Exp. Mol. Pathol. 99, 279–285. 10.1016/j.yexmp.2015.07.011 26216407

[B47] Liu H. Lei C. He Q. Pan Z. Xiao D. Tao Y. (2018). Nuclear Functions of Mammalian MicroRNAs in Gene Regulation, Immunity and Cancer. Mol. Cancer 17, 64. 10.1186/s12943-018-0765-5 29471827 PMC5822656

[B48] Liu H. Wang B. Zhang J. Zhang S. Wang Y. Zhang J. (2017). A Novel Lnc-PCF Promotes the Proliferation of TGF-β1-Activated Epithelial Cells by Targeting miR-344a-5p to Regulate Map3k11 in Pulmonary Fibrosis. Cell Death Dis 8, e3137. 10.1038/cddis.2017.500 29072702 PMC5682666

[B49] Liu M.-W. Su M.-X. Tang D.-Y. Hao L. Xun X.-H. Huang Y.-Q. (2019). Ligustrazin Increases Lung Cell Autophagy and Ameliorates Paraquat-Induced Pulmonary Fibrosis by Inhibiting PI3K/Akt/mTOR and Hedgehog Signalling via Increasing miR-193a Expression. Bmc Pulm. Med. 19, 35. 10.1186/s12890-019-0799-5 30744607 PMC6371511

[B50] Luo Q. Cai Z. Tu J. Ling Y. Wang D. Cai Y. (2019). Total Flavonoids from Smilax Glabra Roxb Blocks Epithelial‐mesenchymal Transition and Inhibits Renal Interstitial Fibrosis by Targeting miR‐21/PTEN Signaling. J. Cel Biochem 120, 3861–3873. 10.1002/jcb.27668 30304552

[B51] Luo X. Zhang D. Xie J. Su Q. He X. Bai R. (2018). MicroRNA-96 Promotes Schistosomiasis Hepatic Fibrosis in Mice by Suppressing Smad7. Mol. Ther. - Methods Clin. Develop. 11, 73–82. 10.1016/j.omtm.2018.10.002 PMC6214875 30406154

[B52] Ma Y. Shi J. Wang F. Li S. Wang J. Zhu C. (2019). MiR‐130b Increases Fibrosis of HMC Cells by Regulating the TGF‐β1 Pathway in Diabetic Nephropathy. J. Cel Biochem 120, 4044–4056. 10.1002/jcb.27688 30260005

[B53] Manickam N. Patel M. Griendling K. K. Gorin Y. Barnes J. L. (2014). RhoA/Rho Kinase Mediates TGF-β1-Induced Kidney Myofibroblast Activation through Poldip2/Nox4-Derived Reactive Oxygen Species. Am. J. Physiology-Renal Physiol. 307, F159–F171. 10.1152/ajprenal.00546.2013 PMC4101629 24872317

[B54] Marquard F. E. Jücker M. (2020). PI3K/AKT/mTOR Signaling as a Molecular Target in Head and Neck Cancer. Biochem. Pharmacol. 172, 113729. 10.1016/j.bcp.2019.113729 31785230

[B55] Meng X.-M. Nikolic-Paterson D. J. Lan H. Y. (2016). TGF-β: the Master Regulator of Fibrosis. Nat. Rev. Nephrol. 12, 325–338. 10.1038/nrneph.2016.48 27108839

[B56] Meng X.-M. Tang P. M.-K. Li J. Lan H. Y. (2015). TGF-Î²/Smad Signaling in Renal Fibrosis. Front. Physiol. 6, 82. 10.3389/fphys.2015.00082 25852569 PMC4365692

[B57] Mi X.-J. Hou J.-G. Jiang S. Liu Z. Tang S. Liu X.-X. (2019). Maltol Mitigates Thioacetamide-Induced Liver Fibrosis through TGF-β1-Mediated Activation of PI3K/Akt Signaling Pathway. J. Agric. Food Chem. 67, 1392–1401. 10.1021/acs.jafc.8b05943 30644744

[B58] O'Shea J. J. Schwartz D. M. Villarino A. V. Gadina M. McInnes I. B. Laurence A. (2015). The JAK-STAT Pathway: Impact on Human Disease and Therapeutic Intervention. Annu. Rev. Med. 66, 311–328. 10.1146/annurev-med-051113-024537 25587654 PMC5634336

[B59] Oh R. S. Haak A. J. Smith K. M. J. Ligresti G. Choi K. M. Xie T. (2018). RNAi Screening Identifies a Mechanosensitive ROCK-JAK2-STAT3 Network central to Myofibroblast Activation. J. Cel Sci 131. 10.1242/jcs.209932 PMC6031327 29678906

[B60] Pannu J. Nakerakanti S. Smith E. Dijke P. T. Trojanowska M. (2007). Transforming Growth Factor-β Receptor Type I-dependent Fibrogenic Gene Program Is Mediated via Activation of Smad1 and ERK1/2 Pathways. J. Biol. Chem. 282, 10405–10413. 10.1074/jbc.M611742200 17317656

[B61] Qin Y. Zhao P. Chen Y. Liu X. Dong H. Zheng W. (2020). Lipopolysaccharide Induces Epithelial-Mesenchymal Transition of Alveolar Epithelial Cells Cocultured with Macrophages Possibly via the JAK2/STAT3 Signaling Pathway. Hum. Exp. Toxicol. 39, 224–234. 10.1177/0960327119881678 31610697

[B62] Ramdas V. McBride M. Denby L. Baker A. H. (2013). Canonical Transforming Growth Factor-β Signaling Regulates Disintegrin Metalloprotease Expression in Experimental Renal Fibrosis via miR-29. Am. J. Pathol. 183, 1885–1896. 10.1016/j.ajpath.2013.08.027 24103556 PMC4188136

[B63] Roderburg C. Luedde M. Vargas Cardenas D. Vucur M. Mollnow T. Zimmermann H. W. (2013). miR-133a Mediates TGF-β-dependent Derepression of Collagen Synthesis in Hepatic Stellate Cells during Liver Fibrosis. J. Hepatol. 58, 736–742. 10.1016/j.jhep.2012.11.022 23183523

[B64] Shen W. Wang Y. Wang D. Zhou H. Zhang H. Li L. (2020). miR-145-5p Attenuates Hypertrophic Scar via Reducing Smad2/Smad3 Expression. Biochem. Biophysical Res. Commun. 521, 1042–1048. 10.1016/j.bbrc.2019.11.040 31732152

[B65] Singh P. Srivastava A. N. Sharma R. Mateen S. Shukla B. Singh A. (2018). Circulating MicroRNA-21 Expression as a Novel Serum Biomarker for Oral Sub-mucous Fibrosis and Oral Squamous Cell Carcinoma. Asian Pac. J. Cancer Prev. 19, 1053–1057. 10.22034/APJCP.2018.19.4.1053 29699056 PMC6031776

[B66] Siomi H. Siomi M. C. (2010). Posttranscriptional Regulation of microRNA Biogenesis in Animals. Mol. Cel 38, 323–332. 10.1016/j.molcel.2010.03.013 20471939

[B67] Solé C. Cortés-Hernández J. Felip M. L. Vidal M. Ordi-Ros J. (2015). miR-29c in Urinary Exosomes as Predictor of Early Renal Fibrosis in Lupus Nephritis. Nephrol. Dial. Transpl. 30, 1488–1496. 10.1093/ndt/gfv128 26040904

[B68] Srivastava S. P. Hedayat A. F. Kanasaki K. Goodwin J. E. (2019). microRNA Crosstalk Influences Epithelial-To-Mesenchymal, Endothelial-To-Mesenchymal, and Macrophage-To-Mesenchymal Transitions in the Kidney. Front. Pharmacol. 10, 904. 10.3389/fphar.2019.00904 31474862 PMC6707424

[B69] Stolzenburg L. R. Wachtel S. Dang H. Harris A. (2016). miR-1343 Attenuates Pathways of Fibrosis by Targeting the TGF-β Receptors. Biochem. J. 473, 245–256. 10.1042/Bj20150821 26542979 PMC4867233

[B70] Sun L. Zhang D. Liu F. Xiang X. Ling G. Xiao L. (2011). Low‐dose Paclitaxel Ameliorates Fibrosis in the Remnant Kidney Model by Down‐regulating miR‐192. J. Pathol. 225, 364–377. 10.1002/path.2961 21984124 PMC3258545

[B71] Tang O. Chen X.-M. Shen S. Hahn M. Pollock C. A. (2013). MiRNA-200b Represses Transforming Growth Factor-β1-Induced EMT and Fibronectin Expression in Kidney Proximal Tubular Cells. Am. J. Physiology-Renal Physiol. 304, F1266–F1273. 10.1152/ajprenal.00302.2012 23408168

[B72] Tao L. Bei Y. Chen P. Lei Z. Fu S. Zhang H. (2016). Crucial Role of miR-433 in Regulating Cardiac Fibrosis. Theranostics 6, 2068–2083. 10.7150/thno.15007 27698941 PMC5039681

[B73] Tao L. Xue D. Shen D. Ma W. Zhang J. Wang X. (2018). MicroRNA-942 Mediates Hepatic Stellate Cell Activation by Regulating BAMBI Expression in Human Liver Fibrosis. Arch. Toxicol. 92, 2935–2946. 10.1007/s00204-018-2278-9 30097701 PMC6590087

[B74] Thatcher J. D. (2010). The TGF- Signal Transduction Pathway. Sci. Signaling 3, tr4. 10.1126/scisignal.3119tr4 20424268

[B75] Valderrama-Carvajal H. Cocolakis E. Lacerte A. Lee E.-H. Krystal G. Ali S. (2002). Activin/TGF-β Induce Apoptosis through Smad-dependent Expression of the Lipid Phosphatase SHIP. Nat. Cel Biol 4, 963–969. 10.1038/ncb885 12447389

[B76] Van der Hauwaert C. Glowacki F. Pottier N. Cauffiez C. (2019). Non-Coding RNAs as New Therapeutic Targets in the Context of Renal Fibrosis. Int. J. Mol. Sci. 20, 1977. 10.3390/ijms20081977 PMC6515288 31018516

[B77] Wang C.-J. Li B.-B. Tan Y.-J. Zhang G.-M. Cheng G.-L. Ren Y.-S. (2020a). MicroRNA-31/184 Is Involved in Transforming Growth Factor-β-Induced Apoptosis in A549 Human Alveolar Adenocarcinoma Cells. Life Sci. 242, 117205. 10.1016/j.lfs.2019.117205 31874165

[B78] Wang J. Chu E. S. H. Chen H.-Y. Man K. Go M. Y. Y. Huang X. R. (2015). microRNA-29b Prevents Liver Fibrosis by Attenuating Hepatic Stellate Cell Activation and Inducing Apoptosis through Targeting PI3K/AKT Pathway. Oncotarget 6, 7325–7338. 10.18632/oncotarget.2621 25356754 PMC4466688

[B79] Wang J. He F. Chen L. Li Q. Jin S. Zheng H. (2018). Resveratrol Inhibits Pulmonary Fibrosis by Regulating miR-21 through MAPK/AP-1 Pathways. Biomed. Pharmacother. 105, 37–44. 10.1016/j.biopha.2018.05.104 29843043

[B80] Wang J. He Q. Han C. Gu H. Jin L. Li Q. (2012a). p53-facilitated miR-199a-3p Regulates Somatic Cell Reprogramming. Stem Cells 30, 1405–1413. 10.1002/stem.1121 22553189

[B81] Wang J. Huang W. Xu R. Nie Y. Cao X. Meng J. (2012b). MicroRNA-24 Regulates Cardiac Fibrosis after Myocardial Infarction. J. Cel. Mol. Med. 16, 2150–2160. 10.1111/j.1582-4934.2012.01523.x PMC3822985 22260784

[B82] Wang J. Jiang C. Li N. Wang F. Xu Y. Shen Z. (2020b). The circEPSTI1/mir-942-5p/LTBP2 axis Regulates the Progression of OSCC in the Background of OSF via EMT and the PI3K/Akt/mTOR Pathway. Cel Death Dis 11, 682. 10.1038/s41419-020-02851-w PMC7443145 32826876

[B83] Wang J. Zhu H. Huang L. Zhu X. Sha J. Li G. (2019a). Nrf2 Signaling Attenuates Epithelial-To-Mesenchymal Transition and Renal Interstitial Fibrosis via PI3K/Akt Signaling Pathways. Exp. Mol. Pathol. 111, 104296. 10.1016/j.yexmp.2019.104296 31449784

[B84] Wang N. Duan L. Ding J. Cao Q. Qian S. Shen H. (2019b). MicroRNA-101 Protects Bladder of BOO from Hypoxia-Induced Fibrosis by Attenuating TGF-β-Smad2/3 Signaling. Iubmb Life 71, 235–243. 10.1002/iub.1968 30549198

[B85] Wang Y. Yang F. Xue J. Zhou X. Luo L. Ma Q. (2017). Antischistosomiasis Liver Fibrosis Effects of Chlorogenic Acid through IL-13/miR-21/Smad7 Signaling Interactions *In Vivo* and *In Vitro* . Antimicrob. Agents Chemother. 61, e01347–16. 10.1128/AAC.01347-16 PMC5278737 27872076

[B86] Wrighton K. H. Lin X. Feng X.-H. (2009). Phospho-control of TGF-β Superfamily Signaling. Cel Res 19, 8–20. 10.1038/cr.2008.327 PMC2929013 19114991

[B87] Wu J. Liu J. Ding Y. Zhu M. Lu K. Zhou J. (2018). MiR-455-3p Suppresses Renal Fibrosis through Repression of ROCK2 Expression in Diabetic Nephropathy. Biochem. Biophysical Res. Commun. 503, 977–983. 10.1016/j.bbrc.2018.06.105 29932921

[B88] Wynn T. A. (2007). Common and Unique Mechanisms Regulate Fibrosis in Various Fibroproliferative Diseases. J. Clin. Invest. 117, 524–529. 10.1172/jci31487 17332879 PMC1804380

[B89] Wynn T. (2008). Cellular and Molecular Mechanisms of Fibrosis. J. Pathol. 214, 199–210. 10.1002/path.2277 18161745 PMC2693329

[B90] Xu Q. Liu Y. Pan H. Xu T. Li Y. Yuan J. (2019). Aberrant Expression of miR-125a-3p Promotes Fibroblast Activation via Fyn/STAT3 Pathway during Silica-Induced Pulmonary Fibrosis. Toxicology 414, 57–67. 10.1016/j.tox.2019.01.007 30658076

[B91] Yamashita M. Fatyol K. Jin C. Wang X. Liu Z. Zhang Y. E. (2008). TRAF6 Mediates Smad-Independent Activation of JNK and P38 by TGF-β. Mol. Cel 31, 918–924. 10.1016/j.molcel.2008.09.002 PMC2621323 18922473

[B92] Yan X. Liu Z. Chen Y. (2009). Regulation of TGF- Signaling by Smad7. Acta Biochim. Biophys. Sinica 41, 263–272. 10.1093/abbs/gmp018 PMC7110000 19352540

[B93] Yang C. Zheng S.-D. Wu H.-J. Chen S.-J. (2016). Regulatory Mechanisms of the Molecular Pathways in Fibrosis Induced by MicroRNAs. Chin. Med. J. (Engl) 129, 2365–2372. 10.4103/0366-6999.190677 27647197 PMC5040024

[B94] Yang F. Luo L. Zhu Z.-D. Zhou X. Wang Y. Xue J. (2017b). Chlorogenic Acid Inhibits Liver Fibrosis by Blocking the miR-21-Regulated TGF-β1/Smad7 Signaling Pathway *In Vitro* and *In Vivo* . Front. Pharmacol. 8, 929. 10.3389/fphar.2017.00929 29311932 PMC5742161

[B95] Yang R. Xu X. Li H. Chen J. Xiang X. Dong Z. (2017a). p53 Induces miR199a-3p to Suppress SOCS7 for STAT3 Activation and Renal Fibrosis in UUO. Sci. Rep. 7, 43409. 10.1038/srep43409 28240316 PMC5327480

[B96] Yang W.-L. Wang J. Chan C.-H. Lee S.-W. Campos A. D. Lamothe B. (2009). The E3 Ligase TRAF6 Regulates Akt Ubiquitination and Activation. Science 325, 1134–1138. 10.1126/science.1175065 19713527 PMC3008763

[B97] Yang Y.-Z. Zhao X.-J. Xu H.-J. Wang S.-C. Pan Y. Wang S.-J. (2019). Magnesium Isoglycyrrhizinate Ameliorates High Fructose-Induced Liver Fibrosis in Rat by Increasing miR-375-3p to Suppress JAK2/STAT3 Pathway and TGF-β1/Smad Signaling. Acta Pharmacol. Sin 40, 879–894. 10.1038/s41401-018-0194-4 30568253 PMC6786319

[B98] Yin Z.-F. Wei Y.-I. Wang X. Wang L.-N. Li X. (2020). Buyang Huanwu Tang Inhibits Cellular Epithelial-To-Mesenchymal Transition by Inhibiting TGF-β1 Activation of PI3K/Akt Signaling Pathway in Pulmonary Fibrosis Model *In Vitro* . BMC Complement. Med. Ther. 20, 13. 10.1186/s12906-019-2807-y 32020862 PMC7076841

[B99] Yu J. S. L. Cui W. (2016). Proliferation, Survival and Metabolism: the Role of PI3K/AKT/mTOR Signalling in Pluripotency and Cell Fate Determination. Development 143, 3050–3060. 10.1242/dev.137075 27578176

[B100] Yuan J. Li P. Pan H. Li Y. Xu Q. Xu T. (2018). miR-542-5p Attenuates Fibroblast Activation by Targeting Integrin α6 in Silica-Induced Pulmonary Fibrosis. Int. J. Mol. Sci. 19, 3717. 10.3390/ijms19123717 PMC6320929 30467286

[B101] Zarjou A. Yang S. Abraham E. Agarwal A. Liu G. (2011). Identification of a microRNA Signature in Renal Fibrosis: Role of miR-21. Am. J. Physiology-Renal Physiol. 301, F793–F801. 10.1152/ajprenal.00273.2011 PMC3191802 21775484

[B102] Zhang Q. Ye H. Xiang F. Song L.-J. Zhou L.-L. Cai P.-C. (2017). miR-18a-5p Inhibits Sub-pleural Pulmonary Fibrosis by Targeting TGF-β Receptor II. Mol. Ther. 25, 728–738. 10.1016/j.ymthe.2016.12.017 28131417 PMC5363213

[B103] Zhang Y. E. (2009). Non-Smad Pathways in TGF-β Signaling. Cel Res 19, 128–139. 10.1038/cr.2008.328 PMC2635127 19114990

[B104] Zhang Y. E. (2017). Non-Smad Signaling Pathways of the TGF-β Family. Cold Spring Harb Perspect. Biol. 9, a022129. 10.1101/cshperspect.a022129 27864313 PMC5287080

[B105] Zhao S. Q. Shen Z. C. Gao B. F. Han P. (2019). microRNA‐206 Overexpression Inhibits Epithelial‐mesenchymal Transition and Glomerulosclerosis in Rats with Chronic Kidney Disease by Inhibiting JAK/STAT Signaling Pathway. J. Cel Biochem 120, 14604–14617. 10.1002/jcb.28722 31148248

[B106] Zhou C. Zeldin Y. Baratz M. E. Kathju S. Satish L. (2019). Investigating the Effects of Pirfenidone on TGF-β1 Stimulated Non-SMAD Signaling Pathways in Dupuytren's Disease -derived Fibroblasts. BMC Musculoskelet. Disord. 20, 135. 10.1186/s12891-019-2486-3 30927912 PMC6441192

